# Cell-free circulating tumor RNAs in plasma as the potential prognostic biomarkers in colorectal cancer

**DOI:** 10.3389/fonc.2023.1134445

**Published:** 2023-04-05

**Authors:** Nana Jin, Chau-Ming Kan, Xiao Meng Pei, Wing Lam Cheung, Simon Siu Man Ng, Heong Ting Wong, Hennie Yuk-Lin Cheng, Wing Wa Leung, Yee Ni Wong, Hin Fung Tsang, Amanda Kit Ching Chan, Yin Kwan Evelyn Wong, William Chi Shing Cho, John Kwok Cheung Chan, William Chi Shing Tai, Ting-Fung Chan, Sze Chuen Cesar Wong, Aldrin Kay-Yuen Yim, Allen Chi-Shing Yu

**Affiliations:** ^1^ R&D, Codex Genetics Limited, Hong Kong, Hong Kong SAR, China; ^2^ Department of Health Technology and Informatics, The Hong Kong Polytechnic University, Hong Kong, Hong Kong SAR, China; ^3^ Department of Applied Biology & Chemical Technology, The Hong Kong Polytechnic University, Hong Kong, Hong Kong SAR, China; ^4^ Department of Surgery, Faculty of Medicine, The Chinese University of Hong Kong, Hong Kong, Hong Kong SAR, China; ^5^ Department of Pathology, Kiang Wu Hospital, Macau, Macau SAR, China; ^6^ Department of Pathology, Queen Elizabeth Hospital, Hong Kong, Hong Kong SAR, China; ^7^ Department of Clinical Oncology, Queen Elizabeth Hospital, Hong Kong, Hong Kong SAR, China; ^8^ School of Life Sciences, The Chinese University of Hong Kong, Hong Kong, Hong Kong SAR, China; ^9^ State Key Laboratory of Agrobiotechnology, The Chinese University of Hong Kong, Hong Kong, Hong Kong SAR, China

**Keywords:** cell-free circulating tumor RNAs, colorectal (colon) cancer, CRC prognostic biomarkers, RNA sequencing (RNA-seq), transcriptome (RNA-seq)

## Abstract

**Background:**

Cell free RNA (cfRNA) contains transcript fragments from multiple cell types, making it useful for cancer detection in clinical settings. However, the pathophysiological origins of cfRNAs in plasma from colorectal cancer (CRC) patients remain unclear.

**Methods:**

To identify the tissue-specific contributions of cfRNAs transcriptomic profile, we used a published single-cell transcriptomics profile to deconvolute cell type abundance among paired plasma samples from CRC patients who underwent tumor-ablative surgery. We further validated the differentially expressed cfRNAs in 5 pairs of CRC tumor samples and adjacent tissue samples as well as 3 additional CRC tumor samples using RNA-sequencing.

**Results:**

The transcriptomic component from intestinal secretory cells was significantly decreased in the in-house post-surgical cfRNA. The *HPGD*, *PACS1*, and *TDP2* expression was consistent across cfRNA and tissue samples. Using the Cancer Genome Atlas (TCGA) CRC datasets, we were able to classify the patients into two groups with significantly different survival outcomes.

**Conclusions:**

The three-gene signature holds promise in applying minimal residual disease (MRD) testing, which involves profiling remnants of cancer cells after or during treatment. Biomarkers identified in the present study need to be validated in a larger cohort of samples in order to ascertain their possible use in early diagnosis of CRC.

## 1 Introduction

Colorectal cancer (CRC) is the third leading cause of cancer-related mortality and morbidity in the world^1^ (1–4). One of the major factors affecting the survival of patients with CRC is the high frequency of recurrence after curative surgery, which is estimated to be 22.5% at 5 years. Approximately 11% of patients survive for 5 years after recurrence (5). Even though advances in cancer therapy have been made in recent decades, metastatic cancer and recurrence still pose a serious threat to the survival of CRC patients (6). Therefore, the identification of post-treatment biomarkers that reflect the potential of CRC recurrence is required to improve the survival of patients.

Genomic alterations associated with oncogenic drivers have traditionally been detected with invasive tissue biopsy, which is highly dependent on the amount of tumor tissue recovered in the biopsy and the initial analysis of the tissue for diagnosis (7). Liquid biopsy, through the use of circulating tumor molecules isolated from blood, has shown to be a promising minimally-invasive approach to detect, monitor, and evaluate the genetic profile of cancer patients (8). Currently, tumor-derived circulating cell-free DNA (ctDNA) analysis has been shown to predict cancer progression. However, there is only a limited amount of ctDNA shed into the circulation, and have different characteristics from patient to patient, which is hard to determine the tumor tissue of origin in cancer patients (9, 10). Although the circulating cfDNA methylation approach in plasma was effective in detecting and localizing cancer with higher specificity (11, 12), these methods may be ineffective without extensive deep sequencing coverage, and their sensitivity and specificity may not be adequate (9, 10). According to our previous study, cfRNA could serve as a potential diagnostic biomarker for patients with colorectal adenoma (13, 14). Therefore, additional circulating cell-free RNA (cfRNA) biomarkers may be required to complement detection by ctDNA to detect cancer, especially at the earliest stages or monitoring the outcome of surgery (10).

Plasma cfRNA is released from cells through active secretion, necrosis, and apoptosis (15, 16). Plasma cfRNA can reflect localized tumor sites as well as systemic tumor responses (17). In this study, we have performed a comprehensive profiling of the transcriptome in both pre-surgical and post-surgical cfRNAs, as well as the paired CRC tumor samples and CRC tumor-adjacent samples, in order to examine the mutational landscape in cfRNAs upon removal of tumor tissue. We deconvolved the relative abundance of cell types in plasma samples using published single-cell RNA-seq datasets and examined whether tissue after surgical might lead to a decrease in the ratio of intestinal cell-associated RNAs in plasma. Novel cfRNA expression biomarkers that showed consistent gene expression changes across in-house plasma samples, tissue samples, and the CRC samples in TCGA were identified. Survival analysis was used to evaluate the prognostic performance of these potential biomarkers and quantitative reverse transcription polymerase chain reaction (qRT-PCR) was conducted to validate these biomarkers in plasma from an independent cohort of 36 cancer patients. The biomarkers we identified could play an important role in the early diagnosis and prognosis of CRC.

## 2 Materials and methods

### 2.1 Subject recruitment

A total of 45 CRC patients were recruited from the Prince of Wales Hospital (PWH) between May 2020 and January 2022 with the approval from the joint Chinese University of Hong Kong- New Territories East Cluster Clinical Research Ethics Committee (CUHK-NTEC CREC; Ref No: 2019.542). Only individuals unrelated to each other were included. Diagnosis of CRC was based on the histological confirmation of colon adenocarcinoma. Patients with hereditary CRC and inflammatory bowel disease were excluded in this study. Each patient was invited to donate tissues (CRC tumor samples and CRC tumor-adjacent samples) and blood (pre-surgery on the day before surgery and post-surgery on the 5^th^-7^th^ day after surgery) for research purposes with written informed consent before the operation. After the surgical removal of the tumor, the tissues were immediately preserved in RNAlater™ Stabilization Solution (Cat# AM7020, Thermo Fisher Scientific, USA) at 4°C overnight in order to make sure the RNAlater can penetrate into the tissue. Then the tissues were stored at -80°C. The tumor-adjacent samples were cut 3 to 4 cm from the tumor. Plasma isolation was performed within 3 hours after the anti-coagulated blood collection using the VACUETTE^®^ TUBE 2 ml K2E K2EDTA (Cat#454024, Greiner Bio-one, Austria). The blood was firstly centrifuged for 1,600 g, 10 minutes at 4 °C. The upper layer plasma without disturbing the buffy coat was collected to the other tube, then re-centrifuged for 16,000 g, 4 °C for 10 minutes to remove residual cell pellet. After that, plasma was collected and preserved by 2 ml TRIzol™ LS Reagent (Cat#10296028, Thermo Fisher Scientific, USA) before storage at −80 °C.

### 2.2 Extraction of cfRNAs from blood

Eight pairs of pre- and post-surgical cfRNA that were prepared for sequencing were extracted from 2-4 ml plasma by using 10ml TRIzol™ LS Reagent (Cat#10296028,Thermo Fisher Scientific, USA). The cfRNA was extracted by using QIAamp cfRNA/cfDNA extraction kit (Cat#55184, Qiagen, Germany) following the manufacturer’s instruction and eluted in 30ul water. The RNA quality was assessed by the TapeStation using High sensitivity RNA assay (Cat#5067-5579, Agilent, USA). The RNA quantity was measured by Qubit™ RNA High Sensitivity (HS) (Cat# Q32852, Invitrogen™, USA) (**Supplementary Table S1**).

### 2.3 Total RNA extraction from tissues

The tissues were shredded by a homogenizer. CRC tumor samples and CRC tumor-adjacent samples from eight patients that were prepared for sequencing were extracted from the AllPrep DNA/RNA kit (Qiagen). The RNA quality was assessed by the TapeStation, using High sensitivity RNA assay (Cat#5067-5579, Agilent, USA). The RINs for all tissue RNA were> 2. The RNA quantity was measured by Qubit™ RNA High Sensitivity (HS) (Cat# Q32852, Invitrogen™, USA).

### 2.4 Ribosomal RNA (rRNA) depletion and library construction for tissue RNA

rRNA depletion was performed on the extracted total RNAs from tissue and subsequent library prep following the NEBNext^®^ rRNA Depletion Kit v2 (Human/Mouse/Rat) (Cat#7400L, New England BioLabs, England)’s protocol, which depletes both mitochondrial (12S and 16S) and cytoplasmic (5S, 5.8S, 18S, and 28S) rRNA species. cDNA synthesis was performed by using Maxima First Strand cDNA Synthesis Kit for RT-qPCR, with dsDNase (Cat#1671, Thermo Scientific™, USA). End-repair, A tailing, adaptor ligation, and library amplification were performed by using the KAPA HyperPlus kit (Cat#KK8512, Rocha, USA). Completed libraries were quantified by each library by Qubit™ 1X dsDNA High Sensitivity (HS) assay kit (Cat#Q33231, Invitrogen™, USA) and the insert size estimation was measured by TapeStation, using D1000 ScreenTape assay (Cat#5067-5582, Agilent, USA).

### 2.5 Library construction for plasma cfRNA

In order to compare the genetic composition of cfRNA before and after surgery, rRNA depletion was not performed in cfRNA as part of the whole transcriptome study (10).

cfRNAs were converted to the cDNA by using SMARTer^®^ Universal Low Input RNA Kit for Sequencing (Cat#634940, Takara Bio, Japan). End-repair, A tailing, adaptor ligation, and library amplification were performed according to the protocol of the NEBNext^®^ Ultra™ II DNA Library Prep Kit for Illumina^®^ (Cat# E7645S, New England BioLabs, England). Completed libraries were quantified by each library by Qubit™ 1X dsDNA High Sensitivity (HS) assay kit (Cat#Q33231, Invitrogen™, USA) and the insert size estimation was measured by TapeStation, using D1000 ScreenTape assay (Cat#5067-5582, Agilent, USA).

### 2.6 RNA sequencing

The Illumina sequencing adaptors were ligated onto the fragments. Constructed libraries were sequenced (300 cycles) using Illumina NextSeq550 (Illumina Inc), according to the manufacturer’s instructions. The Binary Base Call (BCL) files were converted to FASTQ files using the Illumina BCL Convert (v3.7.5). Raw-seq reads quality was assessed using FastQC (v0.11.9)^2^ (18). Adapters and low-quality bases (Q<20 in 4bp sliding window) were trimmed using fastp (v0.20.1) (19). Specifically, seven bases SMARTer adapter from both ends of the reads will be trimmed for plasma cfRNA only. Clean RNA-seq reads were then mapped to the human genome from the Genome Reference Consortium (GRCh38) using STAR aligner (v2.7.7a) with the 2-pass mode (20). Alignments were quantitated using HTSeq (v0.13.5) (21) overlapping with the annotations in GENCODE human release 35. The definition of the biotypes was referenced to GENCODE^3^ (22). Gene expression estimation in terms of Fragments Per Kilobase of transcript per Million mapped reads (FPKM) and differential expression analysis was performed by the R (v4.0.5)/Bioconductor package DESeq2 (v1.30.1) (23). Reactome Pathway Database (24) annotation was performed using the Database for Annotation, Visualization, and Integrated Discovery (DAVID v2021) (25).

### 2.7 Public dataset collections

#### 2.7.1 TCGA dataset

Gene expression data and the corresponding clinical information of 453 patients with CRC (colon adenocarcinoma (COAD) and rectum adenocarcinoma (READ))^4^ were downloaded from the TCGA data portal (26), including 453 CRC tumor samples and 42 CRC tumor-adjacent samples. The identification of the differentially expressed genes (DEGs) was performed using the R/Bioconductor package DESeq2 (v1.30.1).

#### 2.7.2 CRC single-cell datasets

Single-cell 3’ mRNA sequencing data from 23 colorectal cancer patients with the annotation to the cell types including B cell, epithelial cell, mast cell, myeloid cell, stromal cell, and T cell were downloaded from the Gene Expression Omnibus (GEO) database (GSE132465) (27).

#### 2.7.3 Tabula Sapiens

Tabula Sapiens version 1.0 was used to determine the origin of cells of plasma transcriptome. Tabula Sapiens is a human cell atlas of nearly 500,000 cells from 24 organs. The single cell signature used in CIBERSORTx referred to the deconvolution of cell-free RNA tutorial (28) (https://github.com/sevahn/deconvolution/tree/master/deconvolve_cfrna_tutorial).

#### 2.7.4 Cell type abundance determination

The single-cell datasets were used to deconvolute the cell type proportion of bulk tissues and plasma using CIBERSORTx (29). The top 1,000 variated genes in CRC single-cell dataset and Tabula Sapiens dataset were used as the single-cell signatures. All parameters were set as default, except for the permutation was set as 1,000 in the cell fraction imputation step.

#### 2.7.5 Reference-guided *de novo* assemblies

Reference-guided *de novo* assemblies were assembled and quantitated using StringTie (v2.1.4) (30) after mapping to the human genome from the GRCh38 using HISAT2 (v2.2.1) (31), overlapping with the annotations in GENCODE human release 35. Gffcompare (v0.11.2) (32) was used to compare with the reference annotation. The transcripts with the classification code of i, x, y, and u were defined as novel transcripts, otherwise, the transcripts were defined as known transcripts. Differential expression analyses were performed by the R/Bioconductor package ballgown (v2.22.0) (33). The transcripts that (1) not overlapped with regulatory regions in its 5kb upstream and downstream regions from the transcription start site, and (2) with abs(log2Fold-Change) of the expression less than 1 were filtered out as transcripts with low confidence. Regulatory regions were obtained from ORegAnno (v3.0) (34). CPC 2.0 (35) was used to predict the coding potential for the assembled transcripts. AnnoLnc2 (36) was used to predict the expression of the novel transcripts in human samples. We used lncPro to predict the interaction between novel transcripts and proteins (37).

### 2.8 qRT-PCR validation

Plasma samples from 36 patients were used to validate the expression of the candidate genes. The cfRNA was extracted from 1-4.5 ml TRIzol™ LS Reagent (Cat#10296028, Thermo Fisher Scientific, USA) preserved plasma by using miRNeasy Serum/Plasma Kit (Cat#217184, Qiagen, Germany). RNA quantity was measured by Qubit™ RNA High Sensitivity (HS) (Cat# Q32852, Invitrogen™, USA). A majority of the extracted RNAs were below the limit of detection (LOD) of the Qubit™ RNA High Sensitivity (HS) (Cat# Q32852, Invitrogen™, USA) (LOD<10ng) (**Supplementary Table S2**). Reverse transcription reactions were performed following the manufacturer’s instructions using PrimeScript RT Master Mix (Takara) in 10 µL reactions. Otherwise, 30ng RNA was input for reverse transcription.

The primers (**Supplementary Table S3**) for the candidate genes were designed based on the gene sequences gained from the GeneBank, National Centre for Biotechnology Information, NCBI and validated for the absence of self and cross dimers, secondary structures as well as primer efficiency and specificity. Melting curve plot of RT-PCR products showed that no unspecific amplification was detected (**Supplementary Figure S1**).

qRT-PCR assays were performed using the SsoAdvanced Universal SYBR Green Supermix (Cat# 1725270, Bio-Rad, USA) in ABi ViiA7 Real-Time PCR System (ThermoFisher Scientific) in a 20 μL reaction volume according to the manufacturer’s instructions. The thermal cycling condition was 30 seconds at 95°C for initial activation, followed by 45 cycles of 15 seconds at 95°C and 60 seconds at 60°C.

*GAPDH* was demonstrated as useful housekeeping gene to normalize the data, in order to determine the relative target gene expression in cfRNA samples (38). The gene expression was normalized to *GAPDH* among the same patient by delta-delta Ct method as following. The expression level of GAPDH was detected as stable among samples (**Supplementary Figure S2**).


ΔCt=Ct(PACS1/HPGD/TDP2)−Ct(GAPDH)

ΔΔCt=ΔCt−ΔCt(pre−surgical cfRNA)

$$\text{Fold change expression }=2^{-\Delta \Delta \text{Ct}}$$

### 2.9 Survival analysis

451 TCGA CRC samples were split into training and test datasets: 70% of samples of the dataset were randomly selected as training dataset (N=315) and 30% as test dataset (N=136). Gene expression was standardized by removing the mean and scaling to unit variance before analysis. We generated a protective score for each sample – the accumulative weighted gene expression of the *HPGD*, *PACS1*, and *TDP2* by the first principal component. Linear regression was used to fine-tune the protective score. Then samples with a protective score>0.5 were classified as a low-risk group, otherwise as a high-risk group. AUC was used to evaluate the model performance. Survival curves were estimated by the Kaplan-Meier method and compared with a log-rank test.

### 2.10 Statistics

The correlation between gene expression in CRC tumor samples vs TCGA tumor samples and pre-surgical cfRNA vs CRC tumor samples was described using the linear regression model. The significance of the overlapping between significantly upregulated genes in pre-surgical plasma and upregulated protein-coding genes in TCGA tumor samples was described using the hypergeometric test. P values from the Wilcoxon rank-sum method indicated significance levels for differences in cell type proportion across sample groups. Gene expression detected using qRT-PCR was compared between post- and pre-surgical cfRNAs using the paired T-test. The error bars represented mean ± standard deviation (SD).

We used the Python library SciPy (v1.5.2) to perform the statistical analysis. We used adjusted p-value< 0.001 and abs(log2Fold-change) >1 to identify DEGs in in-house CRC tumor samples and CRC tumor-adjacent samples, as well as TCGA tumor samples and tumor-adjacent samples; p-value<0.05 to identify DEGs and DETs in pre- and post-surgical cfRNAs. Gene expression detected using qRT-PCR was compared between post- and pre-surgical cfRNAs using the paired T-test.

### 2.11 Study approval

Each patient was invited to donate tissues (CRC tumor samples and CRC tumor-adjacent samples) and blood (pre-surgical and post-surgical cfRNA) for research purposes with written informed consent before the operation. This study was approved by the joint Chinese University of Hong Kong- New Territories East Cluster Clinical Research Ethics Committee (CUHK-NTEC CREC; Ref No: 2019.542).

### 2.12 Data availability

The raw RNA-seq data of the plasma and tissue samples in this study are available in the NCBI Sequence Read Archive (SRA) database under the accession code PRJNA891435.

## 3 Results

**Figure 1:**
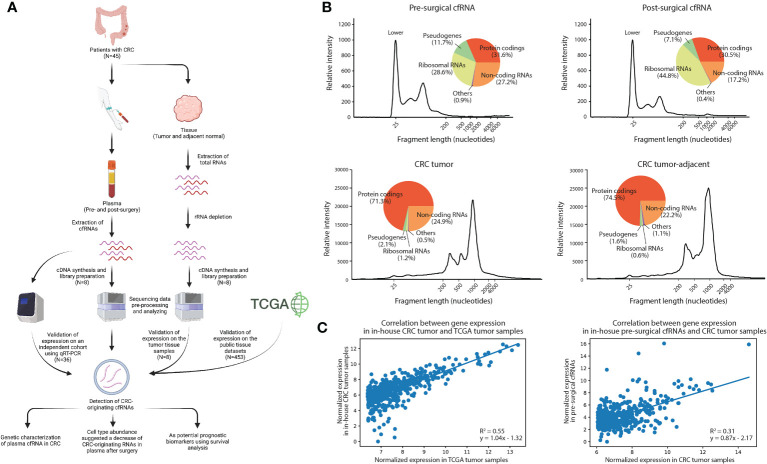
Analytical characterization of cell-free RNA and tissue transcriptome. **(A)** Experimental design of the study, Created with BioRender.com. **(B)** Relative intensity across different fragment lengths of plasma and tissue transcriptomes in a patient with CRC. Pie charts showed the percentage of gene expression in each biotype. **(C)** Correlation between gene expression of the top expressed genes in in-house tumor samples and TCGA datasets, and plasma and tissue datasets. The top 500 expressed genes in TCGA and in-house tissue were selected, respectively.

A total of 45 rectal and colon adenocarcinoma patients were recruited in this study (62.2% men; age: 70.5 ± 8.8 years, **Supplementary Table S4**). cfRNA-seq was performed for 8 patients with matching pre- and post-surgical plasma samples (pre-surgical cfRNA and post-surgical cfRNA) (75% men; 71.6 ± 7.0 years), and bulk RNA-seq was performed using the 5 pairs of CRC tumor samples and CRC tumor-adjacent samples as well as 3 additional CRC tumor samples (87.5% men; age 70.5 ± 5.7 years, **Figure 1A**). The remaining 36 plasma samples (58.3% men; age 70.1 ± 9.3 years) were used in downstream qRT-PCR validation for the biomarkers discovered in this study.

### 3.1 Genetic characterization of plasma cell-free and tissue transcriptome

To characterize the expression landscape of CRC, RNA-seq was performed using the entire yield of extracted cfRNAs and tissue RNAs (see Methods). We systematically profiled the genetic composition of the plasma cell-free and tissue transcriptome (**Figure 1B**; **Supplementary Tables S5**, **S6**). By comparing pre-surgical and post-surgical cfRNAs, we identified that the percentage of noncoding RNAs decreased significantly (*T-test*: p-value=1.42e-03) after surgery (39, 40), while the percentage of rRNAs increased significantly (T-test: p-value=1.20e-02). The genetic composition of tissue, however, has no significant variation between in-house CRC tumor samples and CRC tumor-adjacent samples as expected (**Supplementary Table S6**). This suggests that surgical removal of CRC tissue samples may affect the corresponding cfRNA abundance in plasma.

To examine the level of concordance between in-house CRC tumor sample RNA-seq profiles and published CRC RNA-seq data, we compared the gene expression level between 8 in-house CRC tumor samples and 453 TCGA CRC tumor samples (see Methods). A positive correlation (R^2^ = 0.55, p-value=1.53e-89) was observed in the top 500 expressed genes in the TCGA dataset (**Figure 1C**). Interestingly, the expression between in-house CRC tumor samples and pre-surgical cfRNA was also positively correlated (R^2 ^= 0.31, p-value=9.05e-43). The correlation coefficient is higher than it between in-house CRC tumor-adjacent samples and pre-surgical cfRNA (R^2 ^= 0.23, p-value=1.17e-30), as well as between in-house CRC tumor samples and post-surgical cfRNA (R^2 ^= 0.27, p-value=4.67e-36; **Supplementary Figure S3**). The concordance between the in-house CRC tumor samples and pre-surgical cfRNA leads us to the hypothesis that the patients’ cfRNA could be derived from subpopulations of cells within the tumor (41), additional analysis is therefore necessary to delineate the tissue origin of cfRNA in plasma as to identify biomarkers for CRC in blood.

### 3.2 Cell type abundance suggested a decrease of intestinal cell-originating RNAs in plasma after surgery

**Figure 2:**
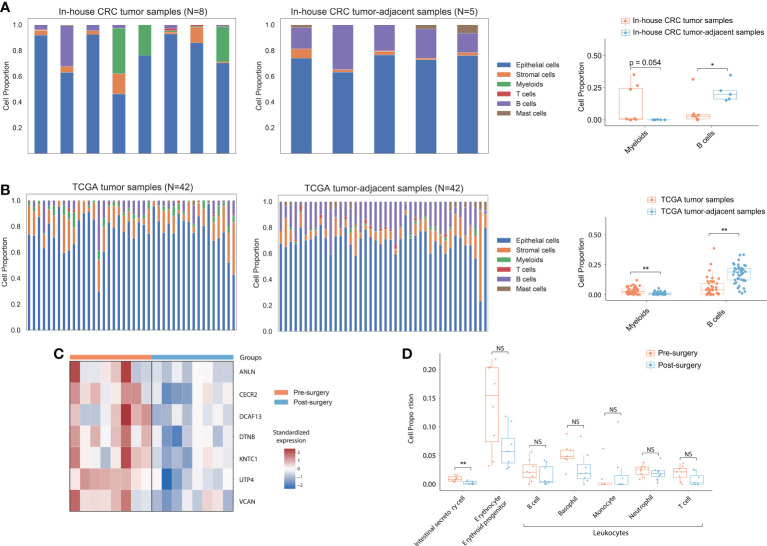
Cell type proportion in tissue and cfRNA. **(A)** Deconvoluted cell type proportion of in-house bulk CRC tumor samples and CRC tumor-adjacent samples. Box plot showed myeloid and B cell fraction distribution from CRC patients. **(B)** Deconvoluted cell type proportion of 42 TCGA bulk tumor and normal samples. Each stacked bar of the left and middle panels represented matched tumor and normal tissue from a single participant. Box plots showed myeloid and B cell fraction distribution from 42 CRC patients in TCGA. **(C)** Heatmap of expression for CRC tissue-highly expressed genes in plasma samples. **(D)** Box plots showed intestinal secretory cells, erythrocytes, erythroid progenitors, and leukocyte fraction distribution from pre- and post-surgical plasma samples. P values from the Wilcoxon rank-sum method indicated significance levels for differences in cell type proportion across sample groups. “**” represented P<0.01; “*” represented P<0.05. NS, not significant.

Given the correlation between in-house CRC tumor samples and pre-surgical cfRNA, we hypothesize that a portion of the cfRNA in plasma could be originating from the cancer tissue. We performed a single-cell deconvolution analysis to predict the relative ratio of contributing cell types based on their specific expression signatures. Firstly, we used CIBERSORTx to predict the cell type proportion of all in-house CRC tumor and CRC tumor-adjacent samples using the published CRC single-cell RNA-seq dataset (GSE132465). A marginal increase in myeloid cells was observed in CRC tumor samples than in CRC tumor-adjacent samples (**Figure 2A**; log2Fold-change=7.35; p-value=5.4e-02), consistent with the role of myeloid cells in providing growth factors and metabolites for tumor growth (42). B Cells, however, were depleted in the tumor samples when compared to CRC tumor-adjacent samples (log2Fold-change=-1.91; p-value=2.3e-02), which is expected for the inhibition role of B cells in tumor development (43). These results were also observed in the TCGA dataset (**Figure 2B**; p-value=2.6e-05 in myeloid cells; p-value=1.1e-08 in B cells) and showed high consistency with the findings from the single-cell RNA-seq data (27).

After demonstrating consistent cell-type specific expression signature between the in-house tissue sample RNA-seq data and TCGA dataset, we hypothesize that the proportion of cfRNA secreted by intestinal cells should be decreased in post-surgical cfRNA samples. Principal component analysis (PCA) based on the top 500 DEGs showed discriminating between the pre- and post-surgical plasma samples (**Supplementary Figure S4**). We detected 50 significantly upregulated genes that expressed across all the samples (FPKM>1) in pre-surgical cfRNA samples, 7 of them overlapped with the 2,379 upregulated protein-coding genes in TCGA tumor samples (**Figure 2C**; Hypergeometric test: p-value=3.63e-03). To determine if the up-regulated genes in pre-surgical cfRNA could be contributed by the intestinal-related cell, we further used the comprehensive human single-cell atlas - Tabula Sapiens (44) to deconvolute the cellular composition of the plasma samples (10). Consistent with the hypothesis, the proportion of intestinal secretory cells was significantly decreased after the surgery (p-value=7.2e-03) when compared to pre-surgical plasma samples (**Figure 2D**). We observed an insignificant change in the expression signature of erythrocyte, erythroid progenitor, and leukocytes between pre- and post-surgical samples (**Figure 2D**), which agrees with a previous study that demonstrated relatively stable expression of these cell types in plasma (28). Taken together, cfRNAs can reflect the intestinal tumor load, which has the potential to be the non-invasive biomarkers for CRC.

### 3.3 Identification of CRC non-invasive differential expression (DE) biomarkers

**Figure 3:**
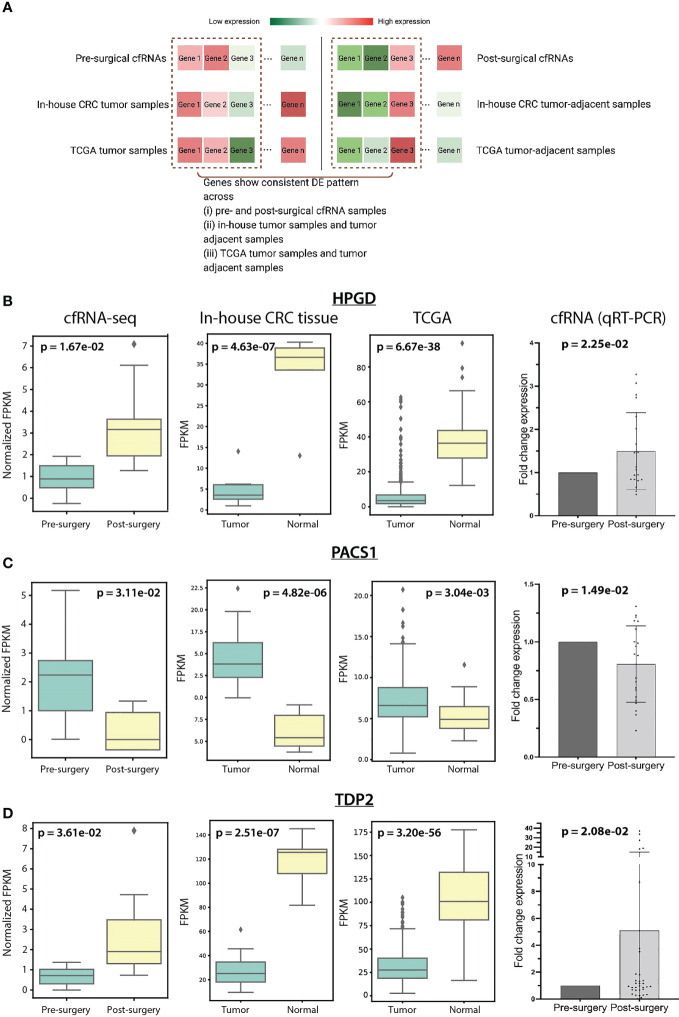
**(A)** Schematic diagram showed the consistent DEGs identification across cfRNAs, in-house CRC tissue samples, and TCGA samples. Gene expression of *HPGD* **(B)**, *PACS1* **(C)**, and *TDP2* **(D)** in cfRNAs, in-house CRC tissue samples, TCGA samples, and in-house qRT-PCR cfRNA data across sample groups.

To identify potential blood-based DE biomarkers for CRC patients, we further performed a *de novo* assembly-based DE analysis to identify transcripts, potentially novel transcripts, that show consistent DE pattern across pre- and post-surgical cfRNA samples, as well as in-house CRC tumor samples and CRC tumor-adjacent samples (**Figure 3A**).

A total of 106,802 transcripts were assembled, the average length of which is 453 bp (see Methods, **Supplementary Figure S5**). We detected 409 differentially expressed transcripts (DETs) with more than 1 exon in the assembled transcripts. 268 out of the 409 transcripts were found to be known transcripts – overlapping with the GENCODE human release 35, and the remaining 141 transcripts were defined as novel transcripts (see Methods). Among the known transcripts, *RNU2-1* which was previously shown to be released from tissue to plasma among CRC patients (45), has been shown to be decreased in the post-surgical cfRNA (**Supplementary Figure S6A**; p-value=0.04, log2Fold-Change=-0.55). A lowered expression was also observed in in-house CRC tumor-adjacent samples (log2Fold-Change=-0.93) and TCGA tumor-adjacent samples (log2Fold-Change=-0.81; **Supplementary Figure S6A**). By selecting highly confident novel transcripts based on fold differences and TSS proximity (34) (see Methods), 10 transcripts were further shortlisted (**Supplementary Table S7**). Interestingly, we identified a significant decrease of the novel *MCF2L-intronic-AS* in post-surgical cfRNA (**Supplementary Figures S6B, C**; p-value=8.31e-03, log2Fold-Change=-1.03) located within the intronic region at antisense strand of *MCF2L*. This novel transcript was predicted as a non-coding transcript with a coding probability of 0.03 by using CPC 2.0 (35) and shown to be expressed in colon adenocarcinoma cell lines by AnnoLnc2 (36). The transcript was predicted to interact with a common set of proteins as *MCF2L-AS1* – a known antisense non-coding RNA of *MCF2L*. *MCF2L-AS1* showed distinctly higher expression in CRC compared to matched normal specimens (46), and its deficiency dramatically impeded cell proliferation, invasion, and migration capacities of CRC (47). *MCF2L-intronic-AS* may serve the consistent role as *MCF2L-AS1* according to interacting with the common proteins. In sum, these significantly depleted post-surgical cfRNAs could be contributed by the reduced intestinal secretory cells after surgical removal of the CRC tissue.

To identify genes with the same DE patterns in (i) CRC tumor tissue and tumor-adjacent tissue and (ii) pre- and post-surgical cfRNA samples (**Figure 3A**), we further performed DE analysis between CRC tumor and tumor-adjacent and identified 1,942 DE genes. Among these 1,942 genes, 11 genes were shown to be overlapping with the 409 DETs in cfRNAs. *CDCA7*, *CELSR3*, *PACS1*, *SNTB1*, and *TBC1D31* showed consistent upregulation in CRC tumor samples and pre-surgical cfRNA, while *GFI1B*, *HPGD*, *SH3BGRL2*, *SIAE*, *PKHD1L1*, and *TDP2* showed downregulation in CRC tumor samples and pre-surgical cfRNA. We further prioritize these genes based on their biomolecular functioning using Reactome Pathway Database (24). Only *HPGD*, *PACS1*, and *TDP2* showed involvement in biological pathways, including metabolism, infection, and DNA repair-related pathways (**Supplementary Table S8**).

### 3.4 Independent external validation of *HPGD*, *PACS1* and *TDP2* expression showed high concordance in CRC

We set out to validate the expression of the three cfRNA biomarkers – *HPGD*, *PACS1*, and *TDP2* identified in our in-house cfRNA and CRC tissue samples using an independent cohort of pre- and post-surgical cfRNA samples (N=36) and published TCGA CRC tumor and tumor-adjacent samples (N=453). *HPGD* has a significantly lower expression in in-house CRC tumor samples (p-value=4.63e-07, log2Fold-Change=-2.70) and pre-surgical cfRNA (p-value=1.67e-02, log2Fold-Change=-0.74). The loss expression of *HPGD* was reported in several colorectal carcinoma cell lines (48) and microscopic colon adenomas (49). We also observed a similarly low *HPGD* expression in TCGA tumor samples (p-value=6.67e-38, log2Fold-Change=-2.86) and the independent in-house pre-surgical cfRNA cohort (p-value=2.25e-02, log2Fold-Change=-0.58) (**Figure 3B**; **Supplementary Table S9**). Especially, the 9 out of 36 patients with N0 stage in the independent in-house cfRNA cohort showed a lower expression in pre-surgical cfRNA (p-value=2.53e-02, log2Fold-Change=-0.61; **Supplementary Figure S7**), implying a role of *HPGD* in early detection of CRC. The expression of *PACS1* and *TDP2* was also examined in both the TCGA CRC RNA-seq data and the independent pre- and post-surgical cfRNA cohort. *PACS1* expression is shown to be consistently higher in both in-house and TCGA CRC tumor samples, as well as pre-surgical cfRNA (**Figure 3C**). *TDP2*, however, is shown to be lowly expressed in the in-house tumor samples, TCGA CRC tumor samples, and pre-surgical cfRNA (**Figure 3D**). In summary, these results confirmed the monitoring potential of *HPGD*, *PACS1*, and *TDP2* in individuals with CRC.

### 3.5 Detection of survival outcome difference in TCGA CRC patients based on the linear combination of *HPGD*, *PACS1*, and *TDP2* expression

**Figure 4:**
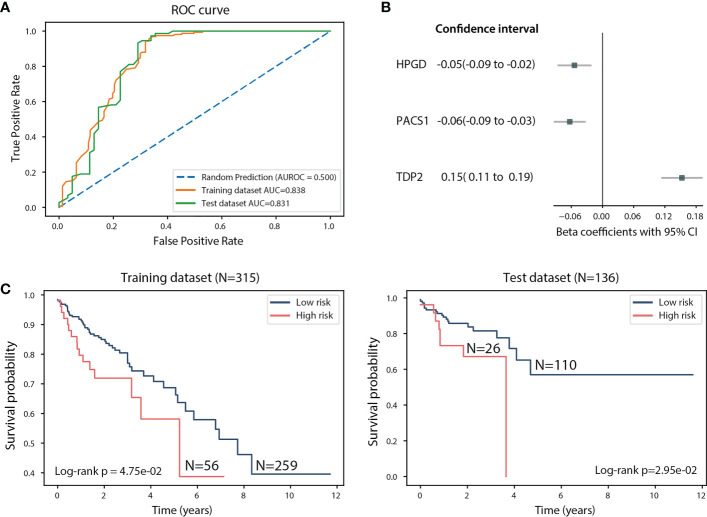
Assessment of the linear regression model using the 451 TCGA CRC samples. **(A)** ROC curve of the training and test datasets. **(B)** Beta coefficients and 95% CI of *HPGD*, *PACS1*, and *TDP2*. **(C)** Kaplan-Meier estimates of overall survival in the training and test datasets according to the linear combination of *HPGD*, *PACS1*, and *TDP2* expression.

We next explored whether the expression of *HPGD*, *PACS1* and *TDP2* can guide the patient classification based on their survival time. We used a linear regression model to investigate the association between the survival time of TCGA CRC patients and the expression of *HPGD*, *PACS1*, and *TDP2* (see Methods). In order to evaluate the fitted model’s accuracy in predicting the risk for CRC patients, we randomly split the TCGA CRC dataset into training (N=315) and test datasets (N=136) and used the receiver operating characteristic (ROC) and the area under the curve (AUC) to assess the model performance (see Methods). The AUC for the training dataset is 0.838 and 0.831 for the test dataset, indicating the good performance of the model (**Figure 4A**). *HPGD* (beta coefficients = -0.05, 95% confidence interval (CI): -0.09 to -0.02, p = 1.25e-03) and *PACS1* (beta coefficients=-0.06, 95% CI: -0.09 to -0.03, p = 6.34e-05) were identified as significant risk factors in the model, while *TDP2* (beta coefficients = 0.15, 95% CI: 0.11 to 0.19, p=3.61e-13) as a significant protective factor (**Figure 4B**). The linear combination of *HPGD*, *PACS1* and *TDP2* expression was used to assess patient survival probability (see Methods). A significant difference was detected for the training dataset (Log-rank p-value: 4.75e-02) and test dataset (Log-rank p-value: 2.95e-02) (**Figure 4C**). The median survival time for a low-risk group (N=110) in the test dataset was 1.65 years compared to 1.36 years for the high-risk group (N=26) (**Figure 4C**). Taken together, the linear combination of *HPGD*, *PACS1*, and *TDP2* expression showed an association with the survival probability of the CRC patient, suggesting the prognostic ability of these potential biomarkers.

## 4 Discussion

Identifying blood-based prognostic markers for minimally invasive cancer detection has been a major focus in the diagnostic area. ctDNA profiling is now being routinely applied clinically for both companion diagnosis and screening for minimal residual disease (MRD) among cancer patients. However, the detection of ctDNA for MRD is challenging as only a minute amount of ctDNA are present in blood at earlier cancer stages, especially in post-surgical setting (7, 10, 50). The cfDNA concentration may fall below the detection limit of the NGS-based ctDNA test, resulting in a very low or even zero mutation allele frequency (MAF) for the mutations (51). More importantly, it is difficult to determine the tumor tissue of origin (TOO) in cancer patients and differentiate informative cfDNA mutations from benign variants such as clonal hematopoiesis (7). Therefore through the amplification of tumor-derived RNA signal, we have shown that the detection of the expressed cfRNA in blood is technically feasible and may help circumvent the existing limitation in ctDNA detection, which will increase cancer detection sensitivity (7, 10). To our knowledge, this is the first study that compared the plasma transcriptomes derived both pre-operatively and post-operatively. Together with paired transcriptome derived from tumor tissues and adjacent normal tissues from CRC patients, we investigated the transcriptional landscape in both blood and tissue upon the surgical removal of CRC tissue.

Previous studies have shown that the cellular components within the tumor immune microenvironment (TIM) are important regulators of primary tumor progression, organ-specific metastasis, as well as a therapeutic response (52, 53). By using published CRC and healthy individual single-cell RNA-seq profiles, we showed that the CRC tumor micro-environment has a marked surge of immune cells, including both myeloid cells and B cells. This agrees with the finding that tumor-infiltrating cells play a critical role in tumor development and treatment response (53), myeloid cells were also previously found to be abundantly present within the TIM among immune cells (54). Interestingly, when examining the cell type contribution among the cfRNA transcriptome profiles, we detected more intestinal secretory cell signatures in pre-surgical cfRNA than post-surgical cfRNA, which have only been reported in the CRC tumor tissue in the previous study (55).

Three significant cfRNA biomarkers *HPGD*, *PACS1*, and *TDP2* were identified through our comprehensive analysis and qRT-PCR validation experiments. The reduction of *HPGD* promotes the expression of *COX-2*, including Ras-activated protein kinase (MAPK) and extracellular signal-regulated kinase (ERK) (56, 57), phosphoinositide 3-kinase (PI3K)–Akt signaling, epidermal growth factor receptor (EGFR) (58) and Wnt/β-catenin (59). The *PACS-1* promotes chromatin organization by increasing the acetylation of chromatin (60) and its deficiency results in replication stress and gross chromosomal aberrations (56). *TDP2* is a DNA repair enzyme that regulates DNA topology by creating double-strand breakage with free 5’ phosphate for re-ligation (61–63). Since *HPGD* is a tumor suppressor gene, as expected, it is freshly expressed in the normal colonic mucosa (59). Interestingly, *HPGD* showed as a significant risk factor in the linear regression model when its expression combined with *PACS1* and *TDP2* expression. Importantly, the model based on the expression of the three genes showed a high AUC (>0.83) of the ROC curve in both training and test datasets. Taken together, linear combination of *HPGD*, *PACS1*, and *TDP2* expression was associated with survival probability, which provides support evidence to potential prognostic biomarkers for CRC. Surprisingly, our research identified a significant decline in *MCF2L-intronic-AS* expression following surgery, which is identical to *MCF2L-AS1* expression. Because potentially interact with the common proteins, *MCF2L-intronic-AS* may play a role in regulating the progression of CRC, which may include promoting cell proliferation, migration, invasion, epithelial-to-mesenchymal transition (EMT), and cell apoptosis (46, 47, 64). There are no studies that have reported the presence of *MCF2L-intronic-AS* in plasma; therefore, further investigations must be conducted to validate the dysregulation pattern of *MCF2L-intronic-AS*.

In the genetic characterization analysis of plasma cfRNA, upregulated expression was observed in rRNA in post-surgical cfRNA samples when compared to pre-surgical cfRNA samples. Two mitochondrially encoded ribosomal RNAs, *MT-RNR2* and *MT-RNR1* are dominant for the increasing expression in the post-surgical cfRNA samples (MT-RNR2: log2FC=2.60, p-value=1.41e-03; MT-RNR1: log2FC=2.73, p-value=1.06e-03), which may play an important role on aiding in the repair of damage during surgery (65). Meanwhile, although no noncoding RNA was observed as a dominant one in the reduction in post-surgical cfRNAs, non-coding RNAs have been reported as drivers of malignant transformation that promote the development of cancers (66). On the other hand, some CRC biomarkers identified from previous studies, such as *CTNNB1* (14), *S100A4* (67), and *EPAS1* (68) were also detected in this study with similar dysregulation patterns, but there was an insufficient sample size in this study that led to these biomarkers being statistically insignificant. While this study shows encouraging results and suggests that the adoption of cfRNA could be useful in a monitoring operation response, future studies with a larger number of replicates per condition should be performed. We acknowledge as a limitation of the present study the small sample size related to cfRNA analysis which did not allow associating the candidate biomarkers to CRC stages as well as investigating on their impact in MRD detection. In conclusion, *HPGD*, *PACS1*, and *TDP2* in CRC plasma samples were demonstrated as potential prognosis biomarkers of CRC, we hope that our results will enable future studies in incorporating cfRNA in the detection, monitoring, and diagnosis of premalignant CRC.

## Data availability statement

The datasets presented in this study can be found in online repositories. The names of the repository/repositories and accession number(s) can be found in the article/**Supplementary Material**.

## Ethics statement

The studies involving human participants were reviewed and approved by The joint Chinese University of Hong Kong- New Territories East Cluster Clinical Research Ethics Committee (CUHK-NTEC CREC; Ref No: 2019.542). The patients/participants provided their written informed consent to participate in this study. Written informed consent was obtained from the individual(s) for the publication of any potentially identifiable images or data included in this article.

## Author contributions

AC-SY, AK-YY, and SCCW designed and supervised the project; NJ performed the bioinformatic analysis; C-MK conducted experiments; XP, WLC, HY-LC, and YKEW performed the qPCR validation; NJ and C-MK wrote the manuscript with input from all authors; SN, WL, and YNW recruited patients and collected samples with consent; HW, HT, AC, WCSC, JC, T-FC, and WST contributed to patient enrolment, provide resource or provide patient samples and scientific advice. All authors contributed to the article and approved the submitted version.

## Footnotes

1
https://seer.cancer.gov/statfacts/html/colorect.html

2
http://www.bioinformatics.babraham.ac.uk/projects/fastqc/

3
https://www.gencodegenes.org/pages/biotypes.html

4
https://portal.gdc.cancer.gov
