# Supplementary material for: Cell-free circulating tumor RNAs in plasma as the potential prognostic biomarkers in colorectal cancer
Source: Front Oncol. 2023 Apr 5;13:1134445. doi: 10.3389/fonc.2023.1134445 (PMC10115432; doi:10.3389/fonc.2023.1134445)
Supplement: Supplementary file 1 [file DataSheet_1.docx]

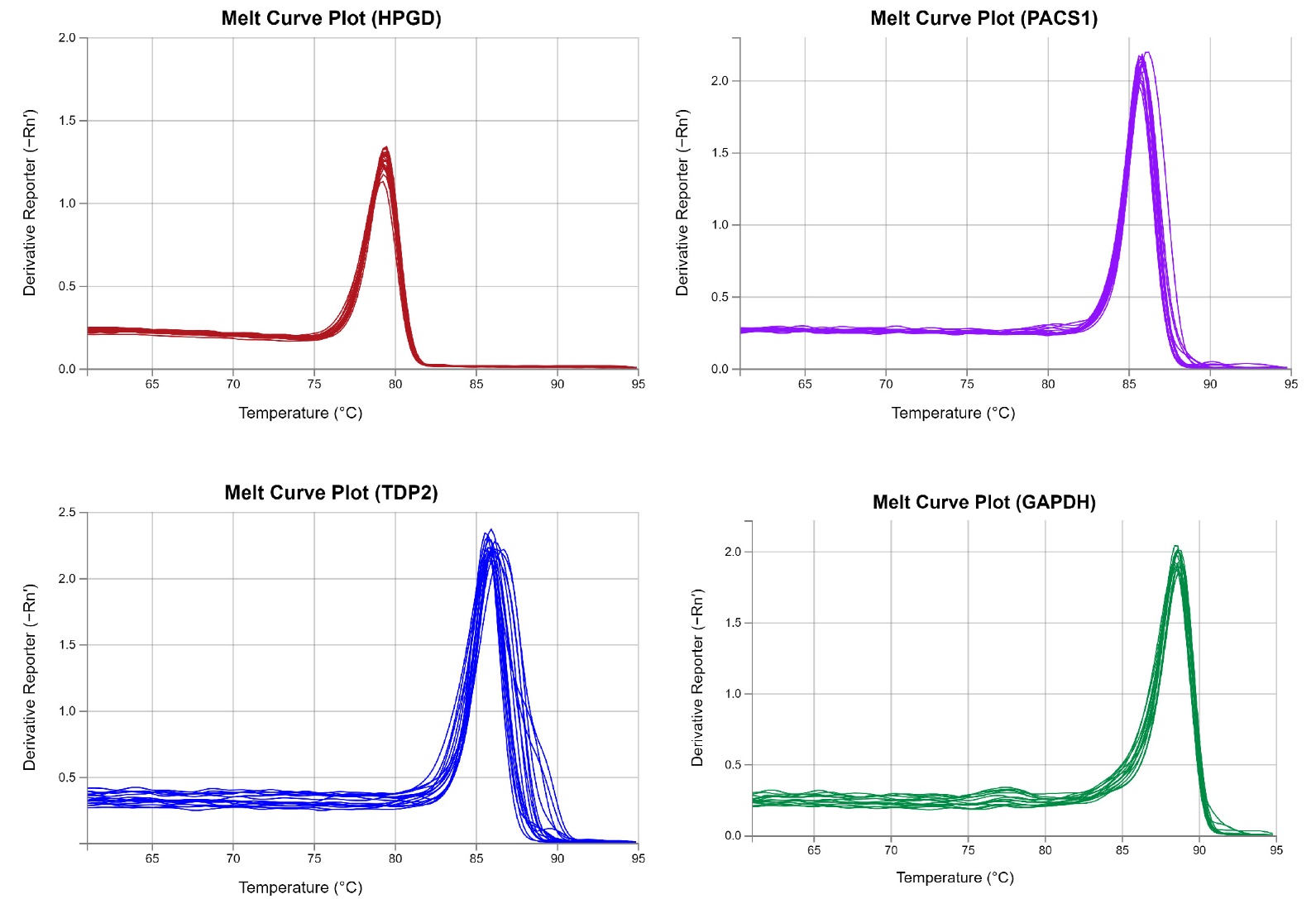


Supplementary Figure S1. Melt curve plot for HPGD, PACS1, TDP2, and GAPDH.


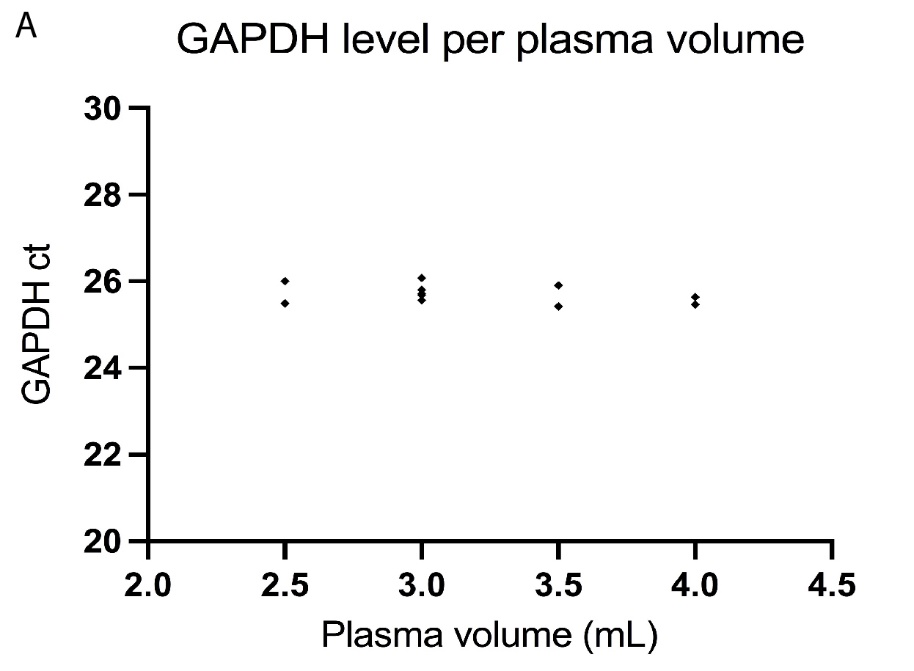


Supplementary Figure S2. The level of *GAPDH* against with different plasma volume using in qPCR validation.


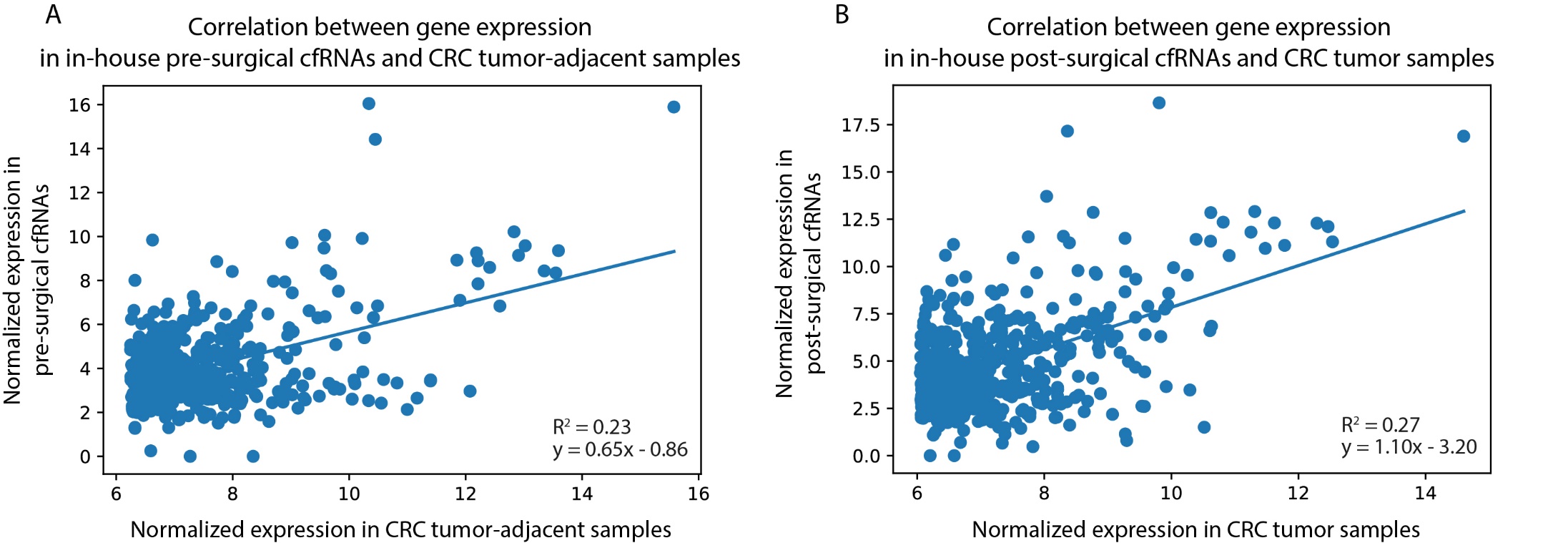


Supplementary Figure S3. Correlation between gene expression of the top expressed genes in in-house plasma and tissue datasets. The top 500 expressed genes in in-house tissue were selected.


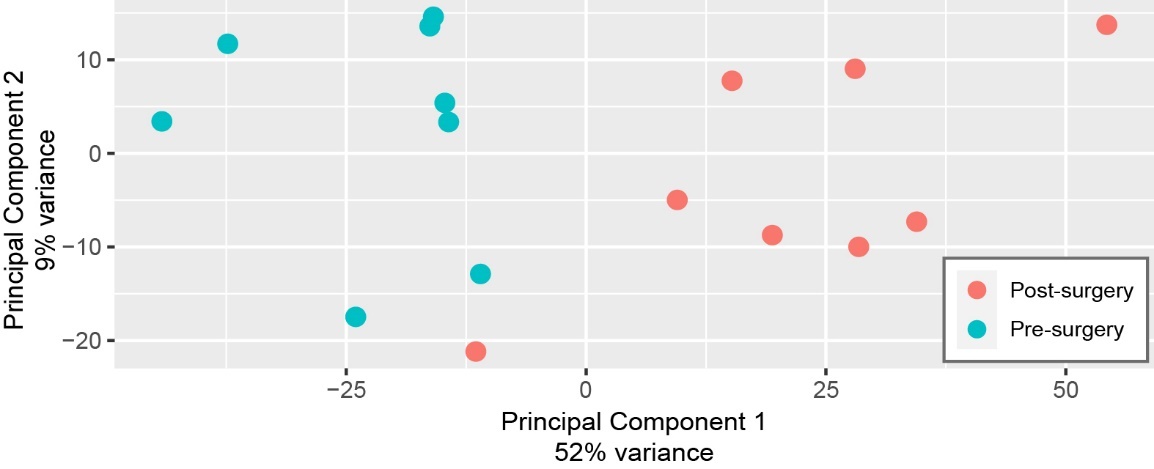


Supplementary Figure S4. Principal component analysis (PCA) using the top 500 DEGs between the pre- and post-surgical plasma samples.


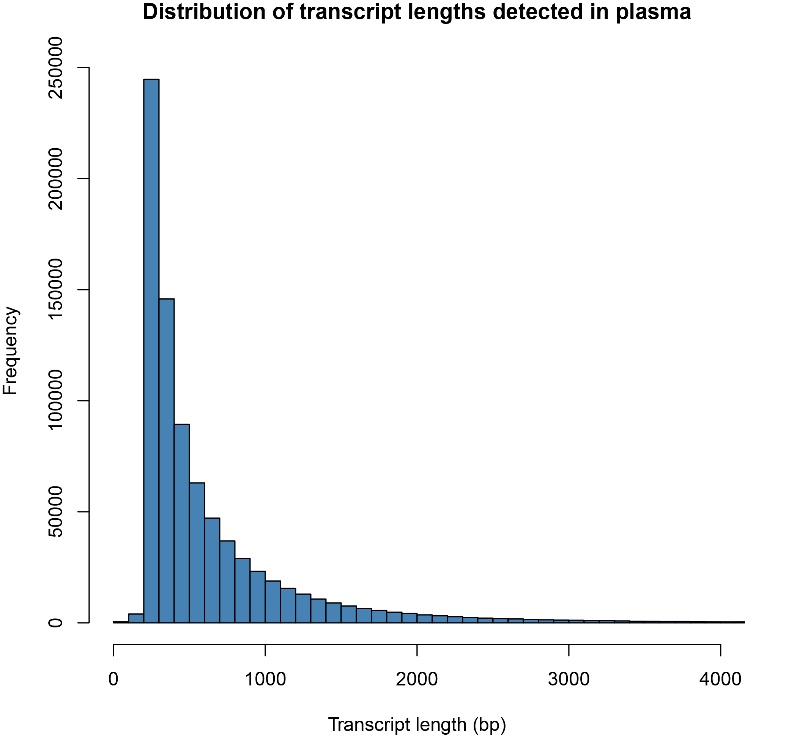


Supplementary Figure S5. Distribution of transcript lengths detected in plasma.


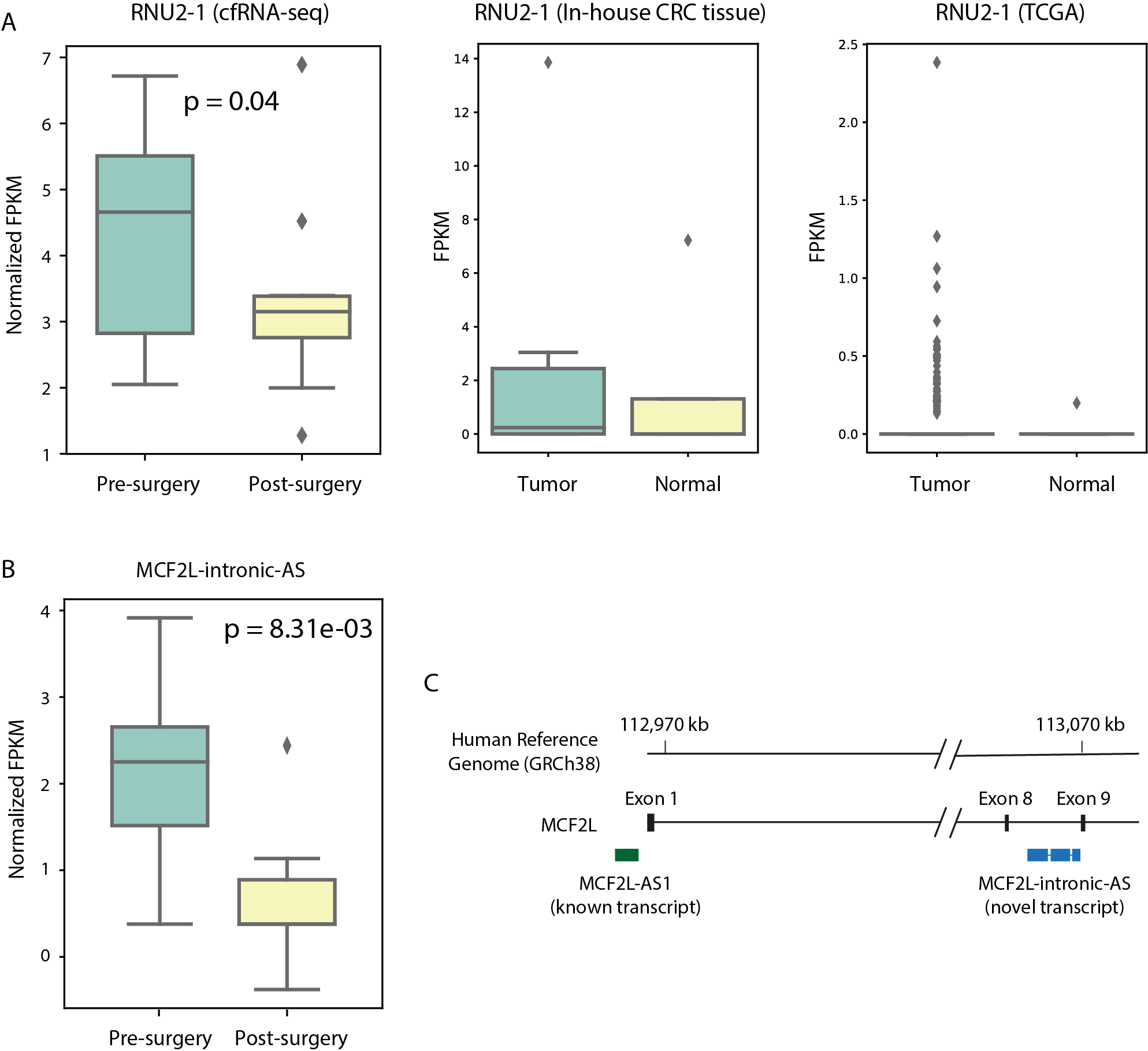


Supplementary Figure S6. (A-B) Box plot showed the expression of RNU2-1 and MCF2L-intronic-AS across sample groups. (C) The genomic locus of MCF2L, known MCF2L-AS1, and the novel detected MCF2L-intronic-AS.


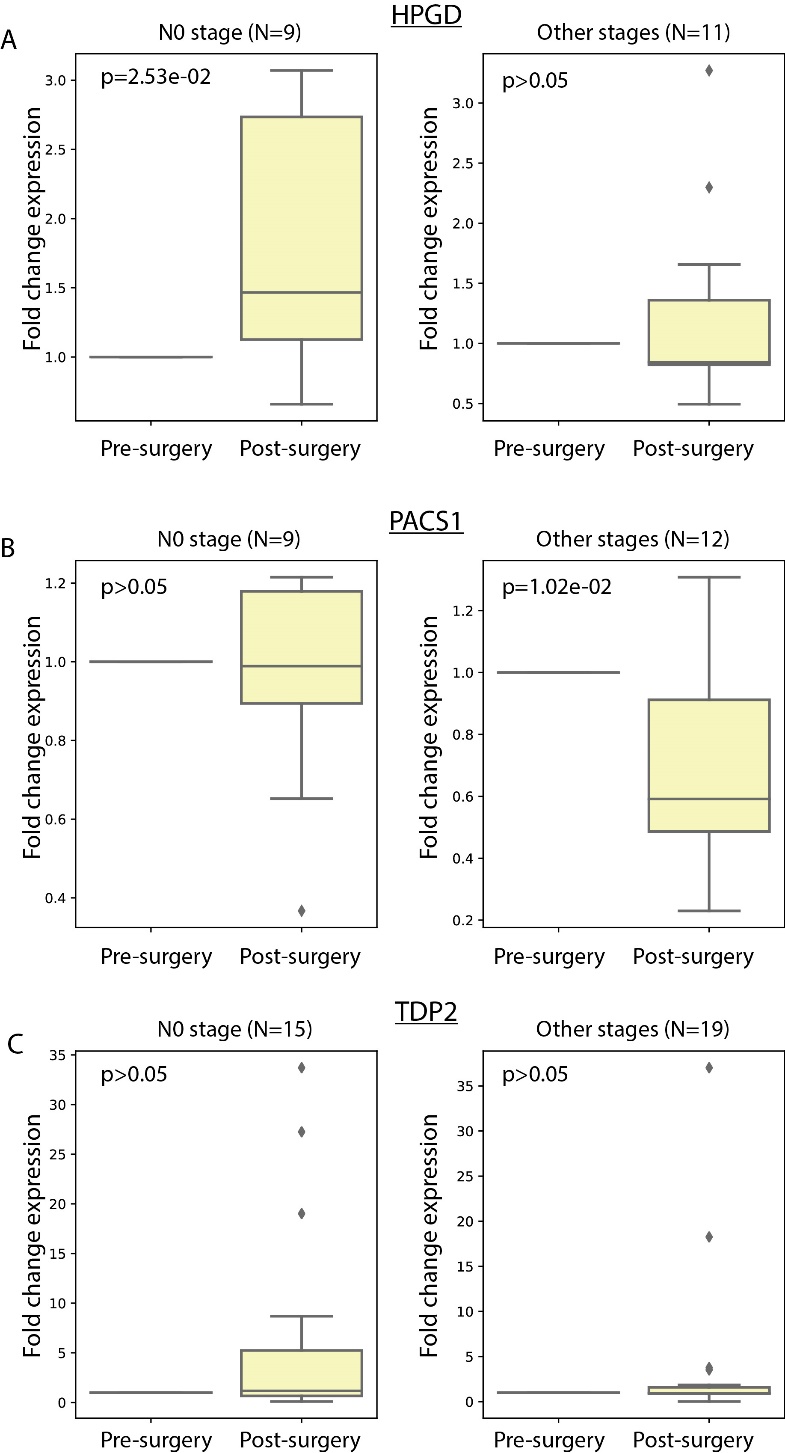


Supplementary Figure S7. Box plot showed expression of HPGD, PACS1, and TDP2 in patients with N0 and other stages respectively in the independent in-house cfRNA cohort.

Supplementary Table S1. The concentration and plasma volume of the RNA-seq cfRNA samples.

|  | Pre-surgery | | Post-surgery | |
| --- | --- | --- | --- | --- |
| Sample | Plasma for extraction (ml) | RNA amount measured by Qubit (ng/uL) | Plasma for extraction (ml) | RNA amount measured by Qubit (ng/uL) |
| PWH 3 | 3 | 0.799 | 3 | 0.238 |
| PWH 11 | 3 | 0.385 | 2 | 0.248 |
| PWH 5 | 3 | 0.459 | 2 | 0.136 |
| PWH 6 | 3 | 0.699 | 4 | 0.244 |
| PWH 7 | 3 | 0.782 | 3 | 0.701 |
| PWH 8 | 3 | 1.39 | 3 | 0.562 |
| PWH 9 | 3 | 0.481 | 3.5 | 0.65 |
| PWH 10 | 3 | 0.477 | 3 | 2.54 |

Supplementary Table S2. The concentration and plasma volume of the qRT-PCR samples.

|  | Pre-surgery | | Post-surgery | |
| --- | --- | --- | --- | --- |
| Sample | Plasma for extraction (ml) | RNA amount measured by Qubit (ng/uL) | Plasma for extraction (ml) | RNA amount measured by Qubit (ng/uL) |
| PWH 13 | 2.5 | <0.2 | 3 | <0.2 |
| PWH 14 | 3.5 | <0.2 | 3.5 | 5.58 |
| PWH 15 | 2.5 | 4.2 | 3.5 | <0.2 |
| PWH 16 | 3 | <0.2 | 3 | <0.2 |
| PWH 17 | 2 | <0.2 | 3.5 | <0.2 |
| PWH 18 | 2.5 | <0.2 | 3 | <0.2 |
| PWH 19 | 2 | <0.2 | 3 | <0.2 |
| PWH 20 | 2.5 | <0.2 | 3.5 | <0.2 |
| PWH 21 | 3 | <0.2 | 3.5 | <0.2 |
| PWH 22 | 2.5 | <0.2 | 2.5 | <0.2 |
| PWH 23 | 3 | <0.2 | 3 | <0.2 |
| PWH 25 | 3 | <0.2 | 3.5 | <0.2 |
| PWH 26 | 3 | 4 | 3 | <0.2 |
| PWH 27 | 3 | <0.2 | 3 | 4.6 |
| PWH 28 | 3 | <0.2 | 2.5 | <0.2 |
| PWH 29 | 3.5 | <0.2 | 3 | <0.2 |
| PWH 30 | 3 | <0.2 | 2 | <0.2 |
| PWH 31 | 3 | 4.6 | 2.5 | <0.2 |
| PWH 32 | 3.5 | <0.2 | 2.5 | <0.2 |
| PWH 33 | 3 | <0.2 | 3 | <0.2 |
| PWH 34 | 3 | <0.2 | 3.5 | <0.2 |
| PWH 35 | 3 | <0.2 | 2.5 | <0.2 |
| PWH 37 | 3.4 | <0.2 | 2.5 | <0.2 |
| PWH 38 | 3.5 | 4.2 | 4 | 5.14 |
| PWH 40 | 2.5 | <0.2 | 3 | <0.2 |
| PWH 41 | 3 | <0.2 | 4.5 | <0.2 |
| PWH 43 | 2.5 | 4.8 | 3 | <0.2 |
| PWH 48 | 2.5 | <0.2 | 3 | <0.2 |
| PWH 49 | 3 | <0.2 | 3 | <0.2 |
| PWH 50 | 2.5 | <0.2 | 3 | <0.2 |
| PWH 51 | 2 | <0.2 | 2.5 | <0.2 |
| PWH 53 | 2.5 | <0.2 | 2.5 | <0.2 |
| PWH 55 | 3 | 4.8 | 2.5 | <0.2 |
| PWH 58 | 2.5 | <0.2 | 2.5 | <0.2 |
| PWH 59 | 4 | 4.6 | 3 | 6.08 |
| PWH 61 | 1 | <0.2 | 1 | <0.2 |

Supplementary Table S3. The primers used for the qRT-PCR validation

| **Gene** | **5´-forward primer-3´ (length / Tm / %GC)** | **5´-reverse primer-3´ (length / Tm / %GC)** | **Product length** |
| --- | --- | --- | --- |
| GAPDH | TGGAAGGACTCATGACCACAGT  (22bp /60.3°C /50%) | TCCCGTTCAGCTCAGGGATG  (20bp / 57.3°C / 60%) | 168 bp |
| PACS1 | AGCCCAAGCTCAAGCCTTTC  (20bp /58.2°C /55%) | GGTGGTGTCTTTTCCGAGGC  (20bp /58.6°C /60%) | 101 bp |
| HPGD | TTTTGGGGGCAGTCAAGGA  (19bp /56.7°C /52.63%) | AGGCTGAACTGTGAATCAAC  (20bp /55.9°C /42.86%) | 114 bp |
| TDP2 | ACGACTGGGAGATGGAAAGGG  (21bp /58.9°C /57.14%) | TAGGTCTTGGGCTCAGAGATGG  (22bp /58.1°C /54.55%) | 103 bp |

Supplementary Table S4. Clinical parameters of each patient

| **Experiment** | **Patient ID** | **Processed sample** | **Gender** | **Age** | **Diagnosis** | **TNM/Dukes stage** | **Histologic type** | **Degree of differentiation** | **KRAS mutation status** | **Adenoma/Polyps** | **Status** | **Chemotherapy** |
| --- | --- | --- | --- | --- | --- | --- | --- | --- | --- | --- | --- | --- |
| RNA-seq | PWH 3 | Pre- and post-surgical cfRNA; tumor | M | 76 | adenocarcinoma | T3N0M0/B | adenocarcinoma | moderate differentiated | unknown | present | Alive | No |
| RNA-seq | PWH 11 | Pre- and post-surgical cfRNA; tumor and tumor-adjacent samples | M | 75 | adenocarcinoma | T4N1M1/D | adenocarcinoma | mucinous | negative | present | Alive | Yes |
| RNA-seq | PWH 5 | Pre- and post-surgical cfRNA; tumor | M | 71 | adenocarcinoma | T1N0M0/A | adenocarcinoma | moderate differentiated | unknown | absence | Alive | No |
| RNA-seq | PWH 6 | Pre- and post-surgical cfRNA; tumor and tumor-adjacent samples | M | 69 | adenocarcinoma | T3N1M0/C | adenocarcinoma | moderate differentiated | unknown | absence | Alive | Yes |
| RNA-seq | PWH 7 | Pre- and post-surgical cfRNA; tumor and tumor-adjacent samples | M | 58 | adenocarcinoma | T3N0M0/B | adenocarcinoma | moderate differentiated | unknown | present | Alive | No |
| RNA-seq | PWH 8 | Pre- and post-surgical cfRNA; tumor and tumor-adjacent samples | F | 69 | adenocarcinoma | T3N1M0/C | adenocarcinoma | moderate differentiated | unknown | present | Alive | Yes |
| RNA-seq | PWH 9 | Pre- and post-surgical cfRNA; tumor | M | 73 | adenocarcinoma | T1N1M0/C | adenocarcinoma | moderate differentiated | unknown | present | Alive | Yes |
| RNA-seq | PWH 10 | Pre- and post-surgical cfRNA | F | 82 | adenocarcinoma | T2N2M0/C | adenocarcinoma | moderate differentiated | unknown | present | Died | Yes |
| RNA-seq | PWH 64 | Tumor and tumor-adjacent samples | M | 73 | adenocarcinoma | T3N0M0/B | adenocarcinoma | moderate differentiated | unknown | absence | Alive | No |
| qRT-PCR | PWH 13 | Pre- and post-surgical cfRNA | F | 73 | adenocarcinoma | T4N2M0/C | adenocarcinoma | moderate differentiated | unknown | absence | Alive | Yes |
| qRT-PCR | PWH 14 | Pre- and post-surgical cfRNA | M | 85 | adenocarcinoma | T3N1M0/C | adenocarcinoma | moderate differentiated | unknown | present | Alive | No |
| qRT-PCR | PWH 15 | Pre- and post-surgical cfRNA | M | 75 | adenocarcinoma | T3N0M0/B | adenocarcinoma | moderate differentiated | unknown | present | Alive | No |
| qRT-PCR | PWH 16 | Pre- and post-surgical cfRNA | F | 71 | adenocarcinoma | T3N0M0/B | adenocarcinoma | moderate differentiated | unknown | absence | Alive | No |
| qRT-PCR | PWH 17 | Pre- and post-surgical cfRNA | F | 66 | adenocarcinoma | T3N2M0/C | adenocarcinoma | moderate differentiated | unknown | absence | Alive | Yes |
| qRT-PCR | PWH 18 | Pre- and post-surgical cfRNA | F | 68 | adenocarcinoma | T4N1M0/C | adenocarcinoma | moderate differentiated | unknown | absence | Alive | Yes |
| qRT-PCR | PWH 19 | Pre- and post-surgical cfRNA | M | 64 | adenocarcinoma | T3N0M0/B | adenocarcinoma | moderate differentiated | unknown | present | Alive | Yes |
| qRT-PCR | PWH 20 | Pre- and post-surgical cfRNA | M | 76 | adenocarcinoma | T3N0M0/B | adenocarcinoma | moderate differentiated | unknown | absence | Died | No |
| qRT-PCR | PWH 21 | Pre- and post-surgical cfRNA | M | 68 | adenocarcinoma | T3N2M1/D | adenocarcinoma | moderate differentiated | unknown | absence | Alive | Yes |
| qRT-PCR | PWH 22 | Pre- and post-surgical cfRNA | M | 65 | adenocarcinoma | T3N0M0/B | adenocarcinoma | moderate differentiated | unknown | absence | Alive | No |
| qRT-PCR | PWH 23 | Pre- and post-surgical cfRNA | M | 74 | adenocarcinoma | T3N0M0/B | adenocarcinoma | moderate differentiated | unknown | present | Alive | No |
| qRT-PCR | PWH 25 | Pre- and post-surgical cfRNA | M | 90 | adenocarcinoma | T3N1M0/C | adenocarcinoma | moderate differentiated | unknown | present | Alive | No |
| qRT-PCR | PWH 26 | Pre- and post-surgical cfRNA | M | 74 | adenocarcinoma | T3N0M0/B | adenocarcinoma | moderate differentiated | unknown | present | Alive | No |
| qRT-PCR | PWH 27 | Pre- and post-surgical cfRNA | M | 60 | adenocarcinoma | T3N0M0/B | adenocarcinoma | moderate differentiated | unknown | absence | Alive | No |
| qRT-PCR | PWH 28 | Pre- and post-surgical cfRNA | M | 69 | adenocarcinoma | T3N1M0/C | adenocarcinoma | moderate differentiated | unknown | absence | Alive | Yes |
| qRT-PCR | PWH 29 | Pre- and post-surgical cfRNA | M | 50 | adenocarcinoma | T3N0M0/B | adenocarcinoma | moderate differentiated | unknown | present | Alive | No |
| qRT-PCR | PWH 30 | Pre- and post-surgical cfRNA | M | 72 | adenocarcinoma | T3N1M0/C | adenocarcinoma | moderate differentiated | unknown | present | Alive | No |
| qRT-PCR | PWH 31 | Pre- and post-surgical cfRNA | F | 64 | adenocarcinoma | T3N1M0/C | adenocarcinoma | moderate differentiated | unknown | present | Alive | Yes |
| qRT-PCR | PWH 32 | Pre- and post-surgical cfRNA | M | 62 | adenocarcinoma | T3N1M0/C | adenocarcinoma | moderate differentiated | unknown | present | Alive | Yes |
| qRT-PCR | PWH 33 | Pre- and post-surgical cfRNA | F | 67 | adenocarcinoma | T3N0M0/B | adenocarcinoma | moderate differentiated | unknown | absence | Alive | No |
| qRT-PCR | PWH 34 | Pre- and post-surgical cfRNA | F | 81 | adenocarcinoma | T4N0M0/B | adenocarcinoma | moderate differentiated | unknown | absence | Alive | No |
| qRT-PCR | PWH 35 | Pre- and post-surgical cfRNA | M | 83 | adenocarcinoma | T3N0M0/B | adenocarcinoma | moderate differentiated | unknown | absence | Alive | No |
| qRT-PCR | PWH 37 | Pre- and post-surgical cfRNA | F | 63 | adenocarcinoma | T3N1M0/C | adenocarcinoma | moderate differentiated | unknown | present | Alive | Yes |
| qRT-PCR | PWH 38 | Pre- and post-surgical cfRNA | F | 76 | adenocarcinoma | T3N1M0/C | adenocarcinoma | moderate differentiated | unknown | present | Alive | Yes |
| qRT-PCR | PWH 40 | Pre- and post-surgical cfRNA | F | 77 | adenocarcinoma | T4N0M1/D | adenocarcinoma | moderate differentiated | negative | absence | Alive | Yes |
| qRT-PCR | PWH 41 | Pre- and post-surgical cfRNA | M | 72 | adenocarcinoma | T3N1M0/C | adenocarcinoma | moderate differentiated | unknown | present | Alive | No |
| qRT-PCR | PWH 43 | Pre- and post-surgical cfRNA | M | 79 | adenocarcinoma | T3N1M0/C | adenocarcinoma | moderate differentiated | positive | present | Alive | No |
| qRT-PCR | PWH 48 | Pre- and post-surgical cfRNA | F | 73 | adenocarcinoma | T3N2M0/C | adenocarcinoma | moderate differentiated | unknown | absence | Died | Yes |
| qRT-PCR | PWH 49 | Pre- and post-surgical cfRNA | F | 61 | adenocarcinoma | T3N1M0/C | adenocarcinoma | moderate differentiated | unknown | absence | Alive | Yes |
| qRT-PCR | PWH 50 | Pre- and post-surgical cfRNA | F | 83 | adenocarcinoma | T3N0M0/B | adenocarcinoma | moderate differentiated | unknown | present | Alive | No |
| qRT-PCR | PWH 51 | Pre- and post-surgical cfRNA | M | 70 | adenocarcinoma | T3N1M0/C | adenocarcinoma | moderate differentiated | unknown | absence | Alive | Yes |
| qRT-PCR | PWH 53 | Pre- and post-surgical cfRNA | M | 63 | adenocarcinoma | T4N0M0/B | adenocarcinoma | moderate differentiated | unknown | present | Alive | No |
| qRT-PCR | PWH 55 | Pre- and post-surgical cfRNA | M | 70 | adenocarcinoma | T3N2M0/C | adenocarcinoma | moderate differentiated | unknown | present | Alive | Yes |
| qRT-PCR | PWH 58 | Pre- and post-surgical cfRNA | M | 64 | adenocarcinoma | T3N0M0/B | adenocarcinoma | moderate differentiated | unknown | present | Alive | No |
| qRT-PCR | PWH 59 | Pre- and post-surgical cfRNA | F | 74 | adenocarcinoma | T3N1M0/C | adenocarcinoma | mucinous | unknown | absence | Alive | Yes |
| qRT-PCR | PWH 61 | Pre- and post-surgical cfRNA | F | 43 | adenocarcinoma | T3N0M0/B | adenocarcinoma | moderate differentiated | unknown | present | Alive | No |

Supplementary Table S5. Genetic composition of pre-surgical and post-surgical cfRNAs.

|  | **Protein codings** | | | | **Ribosomal RNAs** | | | | **Non-coding RNAs** | | | | **Pseudogenes** | | | | **Others** | | | | **Total expression** | |
| --- | --- | --- | --- | --- | --- | --- | --- | --- | --- | --- | --- | --- | --- | --- | --- | --- | --- | --- | --- | --- | --- | --- |
|  | **Pre-surgery expression** | **Pre-surgery %** | **Post-surgery expression** | **Post-surgery %** | **Pre-surgery expression** | **Pre-surgery %** | **Post-surgery expression** | **Post-surgery %** | **Pre-surgery expression** | **Pre-surgery %** | **Post-surgery expression** | **Post-surgery %** | **Pre-surgery expression** | **Pre-surgery %** | **Post-surgery expression** | **Post-surgery %** | **Pre-surgery expression** | **Pre-surgery %** | **Post-surgery expression** | **Post-surgery %** | **Pre-surgery** | **Post-surgery** |
| PWH 3 | 113551.95 | 31.51 | 172361.93 | 29.88 | 53265.79 | 14.78 | 223098.50 | 38.68 | 128139.51 | 35.56 | 125617.20 | 21.78 | 61806.63 | 17.15 | 52785.91 | 9.15 | 3556.79 | 0.99 | 2960.48 | 0.51 | 360320.67 | 576824.02 |
| PWH 11 | 150258.40 | 28.94 | 224308.75 | 23.48 | 192588.90 | 37.09 | 477071.10 | 49.93 | 116475.20 | 22.43 | 218860.34 | 22.91 | 56397.10 | 10.86 | 33625.54 | 3.52 | 3543.86 | 0.68 | 1574.53 | 0.16 | 519263.46 | 955440.27 |
| PWH 5 | 134578.53 | 37.68 | 722217.74 | 18.65 | 39398.52 | 11.03 | 2572204.00 | 66.42 | 119679.53 | 33.51 | 547316.03 | 14.13 | 60211.15 | 16.86 | 29919.04 | 0.77 | 3330.26 | 0.93 | 1265.57 | 0.03 | 357198.00 | 3872922.38 |
| PWH 6 | 148248.94 | 33.84 | 208141.81 | 28.70 | 70897.16 | 16.18 | 303610.40 | 41.86 | 167307.24 | 38.19 | 161972.36 | 22.33 | 49212.85 | 11.23 | 49005.67 | 6.76 | 2432.37 | 0.56 | 2603.57 | 0.36 | 438098.55 | 725333.81 |
| PWH 7 | 145183.41 | 31.63 | 208458.49 | 30.52 | 131478.40 | 28.64 | 306006.70 | 44.80 | 125072.79 | 27.25 | 117251.34 | 17.17 | 53899.10 | 11.74 | 48719.73 | 7.13 | 3395.74 | 0.74 | 2614.34 | 0.38 | 459029.45 | 683050.60 |
| PWH 8 | 108810.92 | 31.60 | 149990.11 | 30.42 | 34888.20 | 10.13 | 149376.50 | 30.30 | 134909.35 | 39.18 | 137645.54 | 27.92 | 62150.49 | 18.05 | 52996.32 | 10.75 | 3591.19 | 1.04 | 2974.81 | 0.60 | 344350.15 | 492983.28 |
| PWH 9 | 153206.55 | 26.35 | 268944.57 | 29.42 | 126346.90 | 21.73 | 425711.30 | 46.57 | 241628.75 | 41.55 | 173327.68 | 18.96 | 57229.21 | 9.84 | 43867.23 | 4.80 | 3056.90 | 0.53 | 2322.47 | 0.25 | 581468.31 | 914173.25 |
| PWH 10 | 150972.97 | 27.48 | 132477.58 | 36.69 | 114853.80 | 20.91 | 42217.81 | 11.69 | 228277.70 | 41.55 | 117086.22 | 32.43 | 52703.37 | 9.59 | 65599.56 | 18.17 | 2571.46 | 0.47 | 3704.43 | 1.03 | 549379.30 | 361085.61 |

Supplementary Table S6. Genetic composition of tissue and T-test between tumor and tumor-adjacent samples.

|  | **Protein codings expression** | **Protein codings %** | **Ribosomal RNAs expression** | **Ribosomal RNAs %** | **Non-coding RNAs expression** | **Non-coding RNAs %** | **Pseudogenes expression** | **Pseudogenes %** | **Others expression** | **Others %** | **Total expression** |
| --- | --- | --- | --- | --- | --- | --- | --- | --- | --- | --- | --- |
| PWH 3 Tumor | 231177.2753 | 86.503748 | 157.622028 | 0.05898 | 26340.04229 | 9.856126 | 9206.330877 | 3.444898 | 364.115352 | 0.136248 | 267245.3858 |
| PWH 11 Tumor | 245068.3394 | 73.961823 | 8302.951086 | 2.505837 | 70394.14587 | 21.24501 | 6935.589963 | 2.093167 | 643.348023 | 0.194163 | 331344.3743 |
| PWH 5 Tumor | 178036.2825 | 89.598096 | 150.329841 | 0.075655 | 13890.43698 | 6.990467 | 6281.546715 | 3.161236 | 346.835012 | 0.174547 | 198705.4311 |
| PWH 6 Tumor | 229406.559 | 73.158458 | 4545.439026 | 1.449555 | 73148.527 | 23.327291 | 4174.212846 | 1.331169 | 2300.157889 | 0.733527 | 313574.8958 |
| PWH 7 Tumor | 255733.9611 | 71.297521 | 4440.649579 | 1.238034 | 89166.79492 | 24.859316 | 7473.185401 | 2.083492 | 1871.034743 | 0.521637 | 358685.6257 |
| PWH 8 Tumor | 216494.3292 | 89.179479 | 77.280506 | 0.031834 | 17266.90044 | 7.112672 | 8416.260227 | 3.46687 | 507.726187 | 0.209145 | 242762.4965 |
| PWH 9 Tumor | 215872.7157 | 89.709194 | 96.405049 | 0.040063 | 16927.29808 | 7.034396 | 7463.985736 | 3.101773 | 275.706795 | 0.114574 | 240636.1114 |
| PWH 64 Tumor | 265490.9094 | 80.378914 | 2296.480319 | 0.695273 | 51647.45374 | 15.636567 | 9586.397322 | 2.902337 | 1277.956894 | 0.386909 | 330299.1977 |
| PWH 11 Tumor-adjacent | 312110.4633 | 82.339885 | 228.865977 | 0.060379 | 55177.18341 | 14.55665 | 10143.77175 | 2.676094 | 1391.087891 | 0.366992 | 379051.3724 |
| PWH 6 Tumor-adjacent | 325238.649 | 79.472755 | 4024.578961 | 0.983414 | 70527.88143 | 17.233638 | 5249.272067 | 1.282671 | 4205.086463 | 1.027522 | 409245.4679 |
| PWH 7 Tumor-adjacent | 318647.4554 | 74.542009 | 2493.472585 | 0.583304 | 94861.06638 | 22.19109 | 6639.418311 | 1.553176 | 4832.249549 | 1.130421 | 427473.6623 |
| PWH 8 Tumor-adjacent | 335496.0765 | 75.709467 | 10893.19453 | 2.458204 | 87895.05657 | 19.834771 | 6036.627732 | 1.362251 | 2815.275273 | 0.635307 | 443136.2306 |
| PWH 64 Tumor-adjacent | 256127.6893 | 86.390937 | 372.653781 | 0.125695 | 31093.03969 | 10.487569 | 7557.664374 | 2.549173 | 1324.137538 | 0.446626 | 296475.1847 |
| T-test between tumor and tumor-adjacent: p-value | - | 6.21e-01 | - | 8.83e-01 | - | 5.54e-01 | - | 7.94e-02 | - | 2.19e-02 | - |

Supplementary Table S7. Genetic characters of the identified high confident transcripts

| **Transcript name** | **Type** | **Gene name** | **chr** | **start** | **end** | **# exons** | **Length** | **Strand** | **Log2fc** | **P val** | **Adjusted p val** |
| --- | --- | --- | --- | --- | --- | --- | --- | --- | --- | --- | --- |
| MSTRG.205985.6 | intronic | MCF2L | chr13 | 113067177 | 113070050 | 3 | 2742 | - | -1.03 | 8.32e-03 | 0.999991443 |
| MSTRG.37144.1 | intergenic | . | chr1 | 121736762 | 121741597 | 2 | 260 | + | 1.21 | 2.15e-02 | 0.999991443 |
| MSTRG.412065.2 | intergenic | . | chr2 | 240308622 | 240310147 | 2 | 1483 | - | 1.80 | 4.96e-02 | 0.999991443 |
| MSTRG.647431.2 | intergenic | . | chr7 | 1271887 | 1273142 | 2 | 1096 | - | -1.06 | 8.07e-03 | 0.999991443 |
| MSTRG.688321.11 | intergenic | . | chr7 | 155345590 | 155349113 | 2 | 2473 | - | -1.20 | 1.50e-03 | 0.999991443 |
| MSTRG.121714.1 | intergenic | . | chr11 | 58770370 | 58786102 | 2 | 1275 | + | 1.03 | 3.74e-02 | 0.999991443 |
| MSTRG.179733.26 | intergenic | . | chr12 | 128241061 | 128242633 | 2 | 1473 | + | 1.20 | 4.52e-02 | 0.999991443 |
| MSTRG.327396.49 | intergenic | . | chr18 | 78512766 | 78515602 | 7 | 2174 | - | -1.02 | 1.43e-02 | 0.999991443 |
| MSTRG.459228.1 | antisense | BTD | chr3 | 15700169 | 15738716 | 2 | 528 | - | -2.23 | 3.09e-03 | 0.999991443 |
| MSTRG.505568.3 | antisense | OPA1 | chr3 | 193647161 | 193683203 | 5 | 661 | - | 1.52 | 2.85e-02 | 0.999991443 |

Supplementary Table S8. Functional annotation of HPGD, PACS1, and TDP2 in Reactome Pathway Database.

| **Gene Name** | **Reactome Pathway** |
| --- | --- |
| HPGD | Metabolism, Synthesis of Lipoxins (LX), Arachidonic acid metabolism, Synthesis of Prostaglandins (PG) and Thromboxanes (TX), Metabolism of lipids, Fatty acid metabolism, Biosynthesis of D-series resolvins, Biosynthesis of DHA-derived SPMs, Biosynthesis of specialized proresolving mediators (SPMs), Biosynthesis of EPA-derived SPMs, Biosynthesis of E-series 18(S)-resolvins |
| PACS1 | HIV Infection, Host Interactions of HIV factors, Disease, Nef-mediates down modulation of cell surface receptors by recruiting them to clathrin adapters, Nef mediated downregulation of MHC class I complex cell surface expression, The role of Nef in HIV-1 replication and disease pathogenesis, Infectious disease |
| TDP2 | DNA Double-Strand Break Repair, Nonhomologous End-Joining (NHEJ), DNA Repair |

Supplementary Table S9. Fold change expression for the independent in-house cfRNA cohort.

| Gene | Sample No. | $2^{-\Delta\Delta Ct}$ in pre-surgical cfRNAs | $2^{-\Delta\Delta Ct}$ in post-surgical cfRNAs |
| --- | --- | --- | --- |
| PACS1 | 13 | 1.000 | 0.686 |
| PACS1 | 15 | 1.000 | 0.652 |
| PACS1 | 21 | 1.000 | 0.230 |
| PACS1 | 22 | 1.000 | 1.179 |
| PACS1 | 25 | 1.000 | 0.521 |
| PACS1 | 26 | 1.000 | 0.367 |
| PACS1 | 27 | 1.000 | 0.894 |
| PACS1 | 28 | 1.000 | 0.468 |
| PACS1 | 30 | 1.000 | 0.400 |
| PACS1 | 31 | 1.000 | 1.308 |
| PACS1 | 33 | 1.000 | 0.989 |
| PACS1 | 34 | 1.000 | 1.183 |
| PACS1 | 35 | 1.000 | 1.110 |
| PACS1 | 37 | 1.000 | 0.583 |
| PACS1 | 41 | 1.000 | 0.600 |
| PACS1 | 43 | 1.000 | 1.231 |
| PACS1 | 48 | 1.000 | 1.001 |
| PACS1 | 50 | 1.000 | 0.962 |
| PACS1 | 53 | 1.000 | 1.215 |
| PACS1 | 55 | 1.000 | 0.492 |
| PACS1 | 59 | 1.000 | 0.882 |
| HPGD | 15 | 1.000 | 0.946 |
| HPGD | 18 | 1.000 | 0.567 |
| HPGD | 20 | 1.000 | 0.659 |
| HPGD | 21 | 1.000 | 0.835 |
| HPGD | 22 | 1.000 | 2.013 |
| HPGD | 23 | 1.000 | 1.466 |
| HPGD | 25 | 1.000 | 0.940 |
| HPGD | 27 | 1.000 | 2.799 |
| HPGD | 30 | 1.000 | 1.657 |
| HPGD | 33 | 1.000 | 1.126 |
| HPGD | 34 | 1.000 | 3.070 |
| HPGD | 37 | 1.000 | 0.811 |
| HPGD | 38 | 1.000 | 0.494 |
| HPGD | 41 | 1.000 | 2.298 |
| HPGD | 43 | 1.000 | 0.843 |
| HPGD | 48 | 1.000 | 1.062 |
| HPGD | 49 | 1.000 | 3.271 |
| HPGD | 50 | 1.000 | 1.458 |
| HPGD | 53 | 1.000 | 2.735 |
| HPGD | 59 | 1.000 | 0.840 |
| TDP2 | 13 | 1.000 | 1.154 |
| TDP2 | 14 | 1.000 | 0.859 |
| TDP2 | 15 | 1.000 | 0.732 |
| TDP2 | 16 | 1.000 | 1.799 |
| TDP2 | 17 | 1.000 | 18.261 |
| TDP2 | 18 | 1.000 | 1.159 |
| TDP2 | 19 | 1.000 | 27.256 |
| TDP2 | 20 | 1.000 | 0.667 |
| TDP2 | 22 | 1.000 | 1.369 |
| TDP2 | 23 | 1.000 | 8.694 |
| TDP2 | 25 | 1.000 | 0.362 |
| TDP2 | 26 | 1.000 | 0.289 |
| TDP2 | 27 | 1.000 | 33.708 |
| TDP2 | 28 | 1.000 | 37.014 |
| TDP2 | 29 | 1.000 | 0.105 |
| TDP2 | 30 | 1.000 | 0.021 |
| TDP2 | 31 | 1.000 | 0.857 |
| TDP2 | 32 | 1.000 | 0.902 |
| TDP2 | 33 | 1.000 | 0.687 |
| TDP2 | 34 | 1.000 | 1.372 |
| TDP2 | 37 | 1.000 | 0.246 |
| TDP2 | 38 | 1.000 | 1.313 |
| TDP2 | 40 | 1.000 | 0.936 |
| TDP2 | 41 | 1.000 | 0.973 |
| TDP2 | 43 | 1.000 | 0.873 |
| TDP2 | 48 | 1.000 | 0.337 |
| TDP2 | 49 | 1.000 | 1.858 |
| TDP2 | 50 | 1.000 | 0.280 |
| TDP2 | 51 | 1.000 | 3.522 |
| TDP2 | 53 | 1.000 | 1.173 |
| TDP2 | 55 | 1.000 | 3.770 |
| TDP2 | 58 | 1.000 | 19.021 |
| TDP2 | 59 | 1.000 | 0.950 |
| TDP2 | 61 | 1.000 | 0.653 |
